# Congenital Long QT Syndrome in Children and Adolescents: A General Overview

**DOI:** 10.3390/children11050582

**Published:** 2024-05-11

**Authors:** Elia Balestra, Marco Bobbo, Marco Cittar, Daniela Chicco, Biancamaria D’Agata Mottolese, Egidio Barbi, Thomas Caiffa

**Affiliations:** 1 Department of Medical, Surgical and Health Sciences, University of Trieste, 34127 Trieste, Italy; egidio.barbi@burlo.trieste.it; 2 Institute for Maternal and Child Health, IRCCS “Burlo Garofolo”, 34127 Trieste, Italy; marco.bobbo@burlo.trieste.it (M.B.); daniela.chicco@burlo.trieste.it (D.C.); biancamaria.dagatamottolese@burlo.trieste.it (B.D.M.); thomas.caiffa@burlo.trieste.it (T.C.); 3 Cardiovascular Department, Centre for Diagnosis and Management of Cardiomyopathies, Azienda Sanitaria Universitaria Integrata di Trieste, University of Trieste, 34127 Trieste, Italy; m.zettar@gmail.com

**Keywords:** congenital long QT syndrome, QTc interval, syncope, palpitations, arrhythmias, β-blockers, implantable cardioverter-defibrillator, left cardiac sympathetic denervation

## Abstract

Congenital long QT syndrome (LQTS) represents a disorder of myocardial repolarization characterized by a prolongation of QTc interval on ECG, which can degenerate into fast polymorphic ventricular arrhythmias. The typical symptoms of LQTS are syncope and palpitations, mainly triggered by adrenergic stimuli, but it can also manifest with cardiac arrest. At least 17 genotypes have been associated with LQTS, with a specific genotype–phenotype relationship described for the three most common subtypes (LQTS1, -2, and -3). β-Blockers are the first-line therapy for LQTS, even if the choice of the appropriate patients needing to be treated may be challenging. In specific cases, interventional measures, such as an implantable cardioverter-defibrillator (ICD) or left cardiac sympathetic denervation (LCSD), are useful. The aim of this review is to highlight the current state-of-the-art knowledge on LQTS, providing an updated picture of possible diagnostic algorithms and therapeutic management.

## 1. Introduction

Congenital long QT syndrome (LQTS) is characterized by a prolongation of heart rate-corrected QT interval (QTc), in the absence of structural cardiopathy or external factors (e.g., drugs, electrolyte abnormalities), which can lead to life-threatening arrhythmias. It generally manifests at pediatric age and represents one of the leading cause of sudden death under 20 years of age [1,2].

There is considerable debate on the current prevalence of this syndrome. Among Caucasians, the prevalence of LQTS is estimated at 1:2000–2500 healthy live births [3]. It is only possible to estimate the prevalence of LQTS, because silent mutation carriers in the general population cannot be detected without genetic screening.

## 2. Materials and Methods

For the aim of this narrative review, we examined the currently available literature. Data were identified through searches of PubMed, UpToDate, and references from relevant articles. We adopted the following inclusion criteria in our literature search: studies published in English on PubMed, Embase, or Web of Science regarding long QT syndrome in children. Literature that did not fulfill these criteria was excluded.

Over 2400 articles were initially found through this search strategy. Selected works were then evaluated by our team, and the most relevant studies were chosen according to the authors’ experience and knowledge.

Reviews, peer-reviewed literature, and guidelines were prioritized, with a focus on works regarding pediatric populations. However, in case of broader concepts valid for both pediatric and adult patients, adult literature was also included. Data from the literature were then integrated with our pediatric experience.

## 3. Pathophysiology

The QT interval represents the time necessary to complete an entire electric cycle in the ventricles, corresponding to the depolarization and repolarization phases of the cardiac action potential.

**Figure 1:**
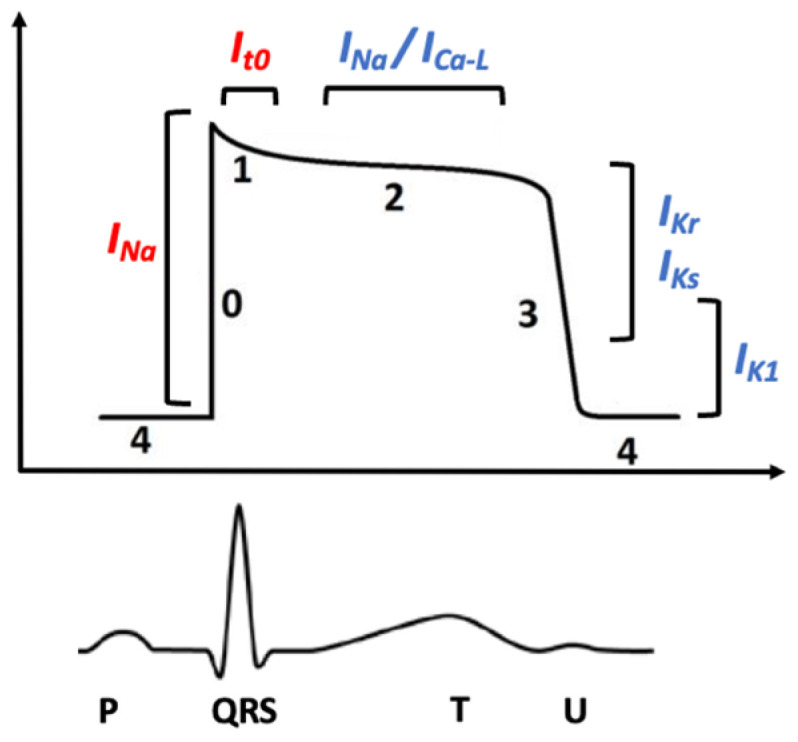
Action potential and electrical currents—Upper figure: inward and outward currents and their relationship with action potential phases; lower figure: electrocardiogram aligned in time with the corresponding action potential. I_Na_—Sodium inward current; I_Ca-L_—Slow calcium inward current; I_Kr_, I_Ks_, I_K1_—Rectifier potassium current; 0—Phase 0 or depolarization phase of action potential; 1—Phase 1 or early repolarization phase of action potential; 2—Phase 2 or plateau phase of action potential; 3—Phase 3 or late repolarization phase of action potential; 4—Phase 4 or resting phase of action potential.

QT prolongation can be sustained by a decreased repolarizing outward K+ current, or by increased depolarizing inward Na or Ca currents (Figure 1) [2].

**Figure 2:**
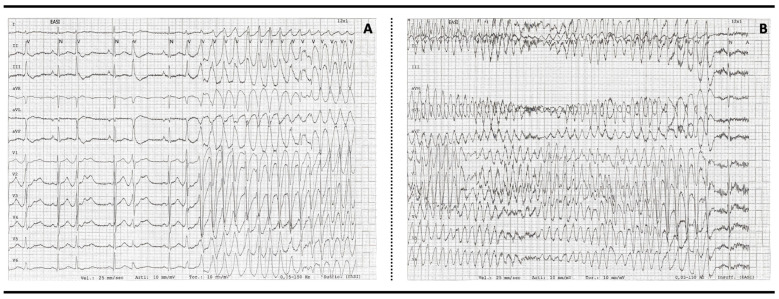
Torsades des pointes and self-limiting ventricular fibrillation. In some cases malignant arrhythmias can be self-limiting; in this case, frequent ventricular extrasystoles are seen, the last of which triggers torsades des pointes (**A**), which degenerates into ventricular fibrillation (**B**) that ends spontaneously.

A severe prolongation of the last part of the ventricular action potential can cause early afterdepolarization; when it reaches the threshold for the consequent fast inward sodium current, it can lead to a triggered beat that can degenerate into fast polymorphic ventricular arrhythmias (such as *torsades des pointes* and ventricular fibrillation), producing syncope, cardiac arrest, or sudden death (Figure 2) [2,4].

## 4. Genotype–Phenotype Correlations and Clinical Presentation

In up to 75% of cases, it is possible to identify an underlying genetic condition affecting genes related to cardiac ion channels’ function. In the remaining 20–25% of cases, genetic analysis does not recognize a specific genotype, although this does not correspond to a lower severity of LQTS [4,5].

Currently, at least 17 genotypes have been associated with LQTS (the main genotypes are presented in Table 1) [1,4,6]. Seven genes have strong evidence for causality, and a well-described specific genotype–phenotype relationship exists for the three most common subtypes (LQTS1, -2, and -3) [6], accounting for almost 90% of positively genotyped cases [7].

This has implications for risk stratification and for the choice of gene-specific treatment.

Underlying genetic variants of the three most common subtypes are represented by the following:

- LQTS1: loss-of-function variants in the potassium channel gene *KCNQ1* (encoding for the α-subunit of the K+ channel Kv7.1, conducting the depolarizing I_Ks_ current), leading to a reduction in the amplitude of the slow delayed rectifier current I_Ks_; as a consequence, the QT interval does not shorten appropriately during tachycardia [2].
- LQTS2: loss-of-function variants in the potassium channel gene *KCNH2* (encoding for the α-subunit of the K+ channel generating the I_Kr_ current), leading to a reduction in the amplitude of the rapid delayed rectifier current I_Kr_.
- LQT3: gain-of-function variants in *SCN5A* (encoding for the α-subunit of the cardiac Na channel and conducting the fast-depolarizing inward sodium current I_Na_), leading to an increase in the amplitude of the late inward sodium current.

**Figure 3:**
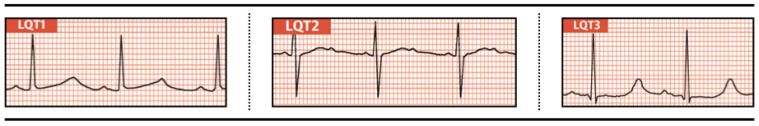
Genotype to electrical phenotype—LQT1: normal T waveform with large base implant; the wave’s amplitude could be low, normal, or high. LQT2: usually low-amplitude T waves with a notched shape (should not to be confused with U waves). LQT3: the main feature is the late onset of the T wave from the isoelectric line; therefore, most of the prolongation is given by the stretch between the Q wave and the beginning of the T wave, rather than by the T wave itself.

The three main LQTS subtypes differ in the following ways (Table 1) [4]:

- Age of onset: younger children in LQTS1; first symptoms around puberty for LQTS2 and LQTS3.
- Triggers for arrhythmic events: adrenergic triggers such as exercise and, in particular, swimming in LQTS1; sudden arousal, especially from sonic stimuli (e.g., sudden noises, phone ringing) or emotional stress, in LQTS2; events in LQTS3 occur more frequently at rest [8]. As regards swimming as a stimulus, it is important to highlight the vagotonic effect caused by the contact of cold water with the face [9].
- Gender at higher risk: male for LQT1; female for LQTS2 and LQTS3.
- ECG pattern (Figure 3): specific morphology of the ST-T segments in V5 (morphologic alterations of ventricular repolarization).
- Sensitivity to K+ serum levels, with LQTS2 patients especially sensitive [2].
- Response to Na channel blockers, which are most effective in LQTS3.

The typical manifestations of LQTS are syncope and palpitations, especially during physical activity or associated with adrenergic stimuli (e.g., strong emotions, sudden noises). Although arrhythmic events can happen from birth in worse cases, initial symptoms most commonly manifest around 11–12 years of age.

The most common subtype of LQTS is represented by autosomal-dominant LQTS in the absence of extra-cardiac involvement. Andersen–Tawil syndrome (LQTS7) [10] and Timothy syndrome (LQTS8) represent forms of autosomal-dominant LQTS associated with extra-cardiac manifestations instead. In detail, other than QT interval prolongation, they present the following characteristics:

- LQTS7 is characterized by ventricular arrhythmias, episodes of flaccid muscle weakness, and facial and corporeal dysmorphism.
- LQTS8 is characterized by cardiac malformations, syndactyly, autism spectrum disorder, and dysmorphisms [11].

In Jervell and Lange-Nielsen syndrome (JLNS), a form of autosomal-recessive LQTS, extreme QT prolongation is combined with congenital deafness [7,12].

## 5. Risk Stratification

LQTS can cause major arrhythmic events or death, and it represents a leading cause of sudden death in populations under 20 years of age [2], with a very high mortality rate in untreated symptomatic patients.

There are differences in risk stratification based on the underlying genetic mutation, clinical aspects, and electrocardiographic features. Jervell and Lange-Nielsen syndrome and Timothy syndrome (LQTS8) represent two of the more malignant subtypes [11,12]. Before puberty, female subjects are at higher risk than males; then, the risk is approximately equal between 13 and 18 years of age, after which it is reversed [13].

Conditions at high risk are represented by the following characteristics:

- QTc > 500 ms (extremely high if QTc > 600 ms).
- Two definitive pathogenic variants and QTc > 500 ms.
- Presence of overt T-wave alternans (direct sign of electrical instability).
- Syncope or cardiac arrest before the age of 7 (related to higher probability of recurrence of arrhythmic events while on β-blockers) or in the first year of life (related to high risk for lethal events).
- Arrhythmic events despite full medical therapy.

Personalized risk stratification can be useful in guiding the choice of the best (medical or interventional) treatment.

## 6. Diagnosis

LQTS can be diagnosed according to QTc prolongation and other specific electrocardiographic findings, the presence of a pathogenic genetic variant, and/or elements from clinical and family history.

Traditionally, the Schwartz score collects electrocardiographic findings, clinical history, and family history (excluding genetic analysis) in order to express the probability of LQTS [14]; in case of a score ≥ 3.5 points, there is a high probability of LQTS. This score is a useful tool to select patients with suspected LQTS who should undergo molecular screening (anyone with a score ≥ 3.0) [2].

More recently, a new algorithm was proposed by the Heart Rhythm Society/European Heart Rhythm Association/Asia Pacific Heart Rhythm Society in their 2013 consensus document. These diagnostic criteria were then confirmed in the 2022 ESC Guidelines, with the modified Schwartz score including genetic findings and excluding congenital deafness. Specifically, LQTS can be diagnosed based on a QTc ≥ 480 ms, with or without symptoms, or a LQTS risk score > 3 (i.e., in case of a pathogenic mutation, diagnosis can be made independent of the QT duration) (Table 2); in case of arrhythmic syncope or cardiac arrest, a QTc ≥ 460 ms is sufficient to consider a diagnosis of LQTS [15,16].

The following must be noted:

Even if prolongation of the QT interval represents the hallmark of LQTS, it is not always present.

A single QTc cannot distinguish all non-LQTS ECGs from all LQTS ECGs, because there can be an overlap of the QTc of individuals with pathogenic variants and normal healthy controls [17]; in fact, approximately 20–25% of the patients with gene-confirmed LQTS may have a normal-range QTc [18,19].

Before diagnosing LQTS on the basis of prolonged QTc, secondary causes of QTc prolongation must be excluded (e.g., drugs, acquired cardiac conditions, electrolyte imbalance).

QTc can be measured through different methods (with different cut-off values), and different formulae have been proposed for heart rate correction [20]. The Bazett method is the most commonly accepted to measure QT length.

### How to Measure the QT Interval

QT interval measurement must be corrected for heart rate of the subject; the so-called Bazett formula (QTc = QT/√RR) allows this and represents the most commonly used formula in studies and clinical practice. Other strategies, such as the Fridericia formula (QTc = QT/RR^1/3^), have been proposed, but their use is more limited [21].

Regarding the Bazett formula, it must be recognized that QTc is underestimated at heart rates lower than 50 beats per minute (bpm), hence why it is recommended to repeat ECG after mild aerobic activity; on the other hand, QTc is overestimated at heart rates higher than 90 bpm (typical for children).

**Figure 4:**
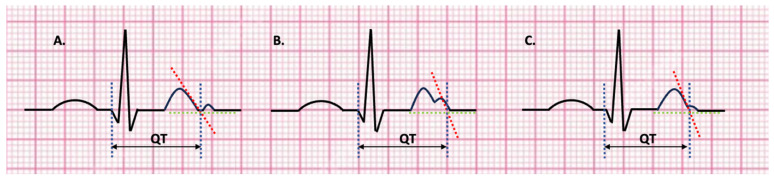
The maximum slope intercept method—The end of the T wave is defined by the intercept between the tangent drawn through the maximum downward slope of the T wave and the isoelectric line. (**A**) U wave is not included in the QT calculation; (**B**) U wave is fused to the T wave, so it’s included in the QT measurement and the tangent is drawn through the second slope; (**C**) The tangent is drawn through the maximum downward slope of the T wave and small U wave is not included in the QT calculation. Green line—isoelectric line; red line—tangent line to the maximum downward slope of the T wave; blue lines—indicating the beginning of QRS and the intercept between the tangent and isoelectric line; QT correspond to the interval between the two blue lines.

Regarding QT interval measurement, there are some general rules to follow:

- Because of the common finding of sinus arrhythmia at pediatric age, average values for the QT interval and RR interval should be used.
- T waves are usually best seen in leads II and V5.
- Several beats should be analyzed and the maximum interval should be considered.
- Low-amplitude U waves should not be included in the QT calculation; in case of large U waves fused to the T wave, they should be included in the calculation (Figure 4A–C) [22].

A rapid movement from lying to an orthostatic position may help with the diagnosis of LQTS, highlighting an inadequate shortening of the QT interval [7,23]. Epinephrine challenge has limited reproducibility and is not recommended as a routine diagnostic tool [7,24].

## 7. Management

### 7.1. Conservative Management

In all patients with LQTS, it is necessary to follow these general rules:

- To avoid QT-prolonging drugs (a list is available on www.qtdrugs.org or www.crediblemeds.org, accessed on 1 March 2024) (class IC recommendation) [16].
- To avoid and adjust electrolyte abnormalities (e.g., during diarrhea, vomiting, metabolic conditions, imbalanced diets) (class IC recommendation) [15,16].
- To avoid strenuous exercise and genotype-specific triggers for arrhythmias (Table 1) [16].

### 7.2. Pharmacological Treatment

The cornerstone of LQTS treatment is represented by ß-blockers. Non-selective ß-blockers, such as nadolol and propranolol, represent the most effective drugs (class IB recommendation) [16,25,26].

ß-Blockers reduce the pro-arrhythmic effects of stress and physical activity, preventing early afterdepolarizations through a blockage of the increase in calcium current led by adrenergic stimuli (or late inward sodium current in the case of propranolol) [4].

β-Blocker therapy causes a significant reduction in the rate of cardiac events and in affected family members [27].

In symptomatic LQTS patients, β-blocker treatment reduced the risk of death from 60% to less than 2% per year in the 10 years since the first arrhythmic episode. Among different LQTS subtypes, LQTS1 patients showed the highest response rate, with the lowest rate of cardiac events, probably related to the antiadrenergic effect of β-blockers on trigger stimuli [28,29].

Even in the absence of studies establishing the most effective dosage, full dosing for age and weight, if tolerated, is recommended: propranolol—2–3 mg/kg/day, with higher doses in very severe cases; nadolol—1–1.5 mg/kg/day divided in 2 doses [2]. Abrupt withdrawal of beta-blocker treatment should be avoided because of the associated increased risk of exacerbation [15].

**Figure 5:**
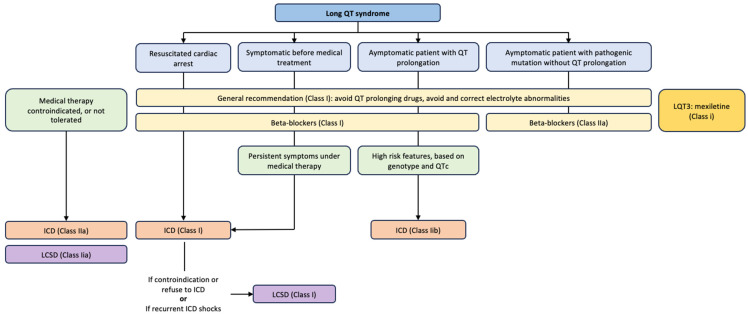
Algorithm for management of long QT syndrome patients (adapted from 2022 ESC Guidelines): ICD—implantable cardioverter-defibrillator; LCSD—left cardiac sympathetic denervation.

According to the ESC 2022 Guidelines (Figure 5), ß-blockers are recommended in patients with a diagnosis of LQTS (class I recommendation) in case of the following:

- Resuscitated cardiac arrest;
- Syncope before starting medical treatment;
- QT prolongation in the absence of symptoms (with or without pathogenic mutation).

These drugs could also be useful in asymptomatic patients with a diagnosis of LQTS, with an underlying pathogenic mutation without QTc prolongation (class IIaB recommendation) [16].

Moreover, as noted previously, therapy is personalized on the basis of risk stratification [30].

The risk of arrhythmia is increased for women with LQTS (especially in case of LQTS2) during pregnancy, and especially in the first year post-partum [31]. The most frequent contraindication to the use of ß-blockers is represented by active asthma, even if treatment is usually well tolerated [32].

Blockers of the late inward sodium current (“sodium channel blockers”) represent another pharmacological possibility (e.g., mexiletine, flecainide and ranolazine) (Figure 4) [4]. Specifically, mexiletine is indicated in subjects with LQTS3 and genetic defects in the SCN5A gene (class IC recommendation), due to its ability to shorten the QTc interval in a specific way [16,33].

There are different degrees of response to mexiletine according to different mutations in SCN5A, and there is no common indication to give mexiletine as a single treatment or in combination with beta-blockers. For these reasons, oral testing should be performed after mexiletine administration to verify a QTc shortening of 40 ms before prescribing chronic treatment [16,33].

In patients with overlapping characteristics of LQTS (QT interval prolongation) and Brugada syndrome (ST segment elevation in leads V1 through V3), related to specific defects of the SCN5A gene, mexiletine does not induce ST segment elevation, unlike flecainide [34]. Moreover, a recent study showed mexiletine’s ability to reduce the QTc in LQTS2 patients [35].

To prevent malignant arrhythmias, it is useful to maintain normal serum K+ levels, avoiding hypokalemia [4], with potassium supplementation particularly effective in LQTS2 (in fact, I_Kr_ strongly depends on the extracellular potassium levels) [36].

A recent preclinical study demonstrated the effectiveness of dual-component suppression-and-replacement (SupRep) KCNQ1 gene therapy in type 1 long QT syndrome, targeting the molecular cause of the disease [37].

### 7.3. Interventional Treatment

#### 7.3.1. ICD—Implantable Cardioverter-Defibrillator [38]

ICD implantation is recommended as secondary prophylaxis, in addition to β-blockers, in patients with a diagnosis of LQTS who have experienced a cardiac arrest (class IB recommendation) [16]. This is due to the fact that there is a high risk of recurrence of arrhythmic events in survivors of a cardiac arrest, even on beta-blockers (14% within 5 years on therapy) [16].

ICD implantation is also recommended in patients with LQTS who are symptomatic despite receiving β-blockers and genotype-specific therapies (Figure 5) [16].

Except under special circumstances, ICD implantation is not indicated in asymptomatic LQTS patients in whom beta-blocker therapy has not been tried (class III recommendation) [15]. ICD implantation may be considered in addition to genotype-specific medical therapies in those asymptomatic patients with a high risk profile (class IIbB recommendation) [16].

ICD implantation must be carefully evaluated for its lifetime implications and possible complications (e.g., inappropriate shocks, infections, displacement related to the physical activity and growth of children), especially in younger patients. In this regards, the “1-2-3 LQTS Risk calculator” represents a recent tool to integrate risk–effectiveness evaluation [39].

There are four different systems of ICD: transvenous systems, typically used in adult age and characterized by a sub-clavicular ICD position and the presence of a transvenous defibrillation lead; epicardial systems, involving epicardial leads for pacing/sensing cardiac rhythm and, in older devices, epicardial defibrillation patches, while in the most recent ones the defibrillation array/coils are implanted in the subcutaneous tissue; the entirely subcutaneous system (S-ICD) is the most recent type of device [40].

Even in the absence of guidelines regarding the relationship between children’s age and weight and the choice of the device, recent works and our clinical experience suggest that epicardial systems should represent the preferred choice in younger children, while transvenous systems or S-ICDs are the most appropriate devices in older/larger children (i.e., those older than 8 years or over 30 kg) [40,41,42].

#### 7.3.2. LCSD—Left Cardiac Sympathetic Denervation

LCSD implies the removal of the first 3–4 thoracic ganglia. It is often effective in reducing the probability of arrhythmic events in high-risk LQTS patients (symptomatic, intolerant of or refractory to beta-blocker treatment, with extremely long QTc) [43].

LCSD is indicated in patients with symptomatic LQTS in case of (a) ICD therapy contraindicated or declined, or (b) multiple shocks or syncopes due to ventricular arrhythmia in a patient with an ICD on β-blockers and genotype-specific drugs (class IC recommendation) (Figure 5) [16].

This procedure can be useful in LQTS patients who experience breakthrough events while on therapy with beta-blockers/ICDs (class IIa recommendation).

If beta-blockers and genotype-specific therapies are not tolerated or are contraindicated at the therapeutic dose in symptomatic LQTS patients, either ICD implantation or LCSD should be considered (class IIaC recommendation) [16].

## 8. Ongoing Discussions

### 8.1. Screening for Long QT Syndrome

Long QT syndrome would appear to be a suitable disease for universal screening because of its relatively high incidence in the general population (1:2000–2500 healthy live births), the fact that sudden cardiac death is a presenting symptom in around 12% of cases [44], and the safety, effectiveness, and cost-effectiveness of treatment with ß-blockers. However, there are some issues and challenges that remain unresolved.

First of all, the quality and the type of the screening test represent the main issue. The standard 12-lead ECG entails the following problems:

It does not recognize LQTS in case of a normal QTc interval.

The Bazett formula can be inaccurate at elevated newborn heart rates.

Measuring QT is time-consuming, operator-dependent, and can be impacted by disturbing factors (e.g., newborns crying).

In this sense, future perspectives could be represented by artificial intelligence, which could have the ability to identify additional features in ECG waveforms in healthy-appearing individuals with underlying LQTS syndrome [45].

On the other hand, genetic testing costs are too high to be considered sustainable for universal screening at the moment.

Another point of interest is the timing of the screening. In fact, the QTc interval’s length increases in the first few months, with a subsequent shortening, and with the highest value between 6 and 11 weeks of age [46].

This period approximately corresponds to the peak period of occurrence of sudden infant death syndrome (SIDS), which, despite having a multi-factorial origin, is attributable to LQTS mutations in nearly 10% of cases [47]. So, one month of age could represent an appropriate time to perform ECG screening for QT prolongation, but this is a challenging time to arrange for a population screening, and further follow-up studies are needed [46]. Before sport participation and school age have also been proposed as ideal timepoints for an ECG screening program. Root mean square (RMS) electrocardiography, a new technique that can give more reliable measures of repolarization, can help from this point of view [44].

In case of sudden death of unknown origin in a family member, it is appropriate to investigate all children who are first-degree relatives with proper medical history, clinical examination, ECG, and echocardiogram, even if these examinations may not be sufficient to diagnose the underlying disease (e.g., a normal ECG in case of LQTS) [48].

### 8.2. “Concealed” Long QT Syndrome

There is a huge debate regarding the need to treat the so-called “silent mutation carriers”, meaning subjects with normal resting QTc values and a genotype-confirmed long QT syndrome. People with this type of “concealed” LQTS represent about 25% of the at-risk LQTS population. Moreover, familial screening of LQTS patients leads to the identification of a large number of asymptomatic carriers of the gene in the general population.

While all patients should be treated with ß-blockers in case of the onset of symptoms, the coach to treat asymptomatic individual is more argued.

The work of Goldenberg et al. [19] highlighted that in individuals with normal-range QTc the frequency of aborted cardiac arrest or sudden cardiac death is significantly lower than in patients with prolonged QTc intervals (4% vs. 15%) but higher than in healthy controls. Risk factors are represented by the location and type of mutation (with higher risk in case of transmembrane missense mutations) and the LQTS genotype (10- and 8-fold increase in the risk of life-threatening events in case of LQTS1 and LQTS3 genotypes, respectively, compared with the LQT2 genotype in normal-QTc individuals). Life-threatening events occur earlier than in patients with concealed LQTS (mostly after 10 years of age) [19].

Being able to identify this subgroup of individuals at higher risk can help to put in place measures to avoid life-threatening LQTS-associated arrhythmias, consisting in avoiding drugs that prolong the QT interval and starting a prophylactic β-blocker treatment.

Waddell-Smith and colleagues tried to identify the pediatric subgroup eligible to take ß-blockers [49]. ß-Blockers were considered to be “not essential” in asymptomatic LQTS carriers if the following conditions are all fulfilled:

- QTc is constantly shorter than 470 ms;
- C-loop missense LQT1 mutation is not present;
- Patient does not take part in high-risk activities (particularly swimming);
- Patient is a male of pre-school age or a female of pre-pubertal age [49].

More generally, the recent 2022 ESC Guidelines [16] suggest to consider ß-blockers in all patients with a pathogenic mutation, even if with a normal QTc interval.

### 8.3. Long QT Syndrome and Sport

First of all, congenital long QT syndrome (LQTS) should be distinguished from acquired forms (i.e., caused by drugs, electrolyte imbalances such as hypokalemia, or hypomagnesemia), in which case sport activity should be avoided until correction of the underlying cause [50].

In case of congenital long QT syndrome, there is indication for disqualification from competitive sport for all athletes with symptoms [50].

In asymptomatic LQTS individuals—the so-called “genotype positive—phenotype negative” subjects, which means mutation carriers with a normal QT interval (i.e., <470/480 ms in men/women)—sport participation must be evaluated with a personalized approach, and the extent of precautionary measures should be considered (class IIaC recommendation) [50]. A negative exercise stress test has no predictive value [51]. In contrast, in case of QTc > 500 ms or QTc > 470/480 ms in men/women with genetically confirmed LQTS, participation in high-intensity sports (both recreational and competitive) is not recommended, even if they are on ß-blocker therapy [50]. In case of prior cardiac arrest or arrhythmic syncope, participation in competitive sports is not recommended [50].

Moreover, while bradycardia can lead to underestimation of QTc length in athletes, on the other hand, intense training can lead to repolarization changes due to mechanical stress on the ventricles, indistinguishable from LQTS. However, this type of QTc prolongation is reversible with 3–4 months of detraining. For this reason, in case of an asymptomatic athlete without family history and without a predisposing genotype, the Italian Guidelines suggest a complete re-evaluation in a referral center (with Holter and exercise test) after a period of detraining [52].

In fact, in Italy, all athletes with LQTS are disqualified from competitive sport apart from asymptomatic individuals with a positive genotype and a negative or borderline phenotype, on treatment with ß-blockers. In these subjects, eligibility may be considered according to a personalized approach, taking into account the type of sport activity, aspects related to current drug therapy, the type of genetic mutation, family history, and QTc values [52].

### 8.4. Drug-Induced QT Prolongation

Drug-induced LQTS represents a concerning issue for the clinician. Its occurrence is unpredictable, even if most patients often present at least one other risk factor for QT interval prolongation.

Higher doses of QT-prolonging drugs, combinations of them, or pharmacokinetic or pharmacodynamic drug interactions increase the risk of drug-induced LQTS.

If a child takes more than one drug affecting the QT interval or presents risk factors for developing QT prolongation, it is necessary to perform an ECG, to correct the risk factors (if possible), and to evaluate the administration of a second-choice agent before starting a QT-prolonging treatment.

The most commonly used pediatric agents associated with drug-induced LQTS are represented by macrolides (azithromycin, clarithromycin), fluoroquinolones (ciprofloxacin, moxifloxacin, levofloxacin), and azole antifungals (fluconazole). Other agents, which should be avoided in case of congenital LQTS, include trimethoprim/sulfamethoxazole, antipsychotic drugs (chlorpromazine, haloperidol, clozapine), anti-vomiting drugs (domperidone, ondansentron), some anesthetics (propofol, sevoflurane), flecainide, sulpiride, and tacrolimus. A more detailed list is available at www.qtdrugs.org or www.crediblemeds.org (accessed on 1 March 2024).

In case of drug-induced LQTS, the first step is to discontinue any drugs and to resolve any correctable risk factors. Most *torsades de pointes* episodes resolve spontaneously, but in case of persistent arrhythmia intravenous magnesium sulfate should be administered [53].

## 9. Conclusions

In this review, we provide an overview on the current state-of-the-art knowledge on LQTS. A practical and easily understandable approach to diagnosis and treatment is presented. Most recent evidence in the literature was selected to define the diagnostic and therapeutic algorithms. Finally, ongoing discussions on LQTS are brought to the readers’ attention, with the awareness of the continuous changes and of the need for further studies to solve the remaining open questions.

## Figures and Tables

**Table 1:** Main LQTS genotypes: characteristics and features.

	LQTS1	LQTS2	LQTS3	LQTS4	LQTS5	LQTS6	LQTS7	LQTS8
**Gene mutation**	*KCNQ1*	*KCNH2*	*SCN5a*	*ANK2*	*KCNE1*	*KCNE2*	*KCNJ2*	*CACNA1C*
**Effect on current**	I_Ks_—LoF	I_Kr_—LoF	I_Na-L_—GoF	I_Na_—GoF LoF ankyrin-B	I_Ks_—LoF	I_Kr_—LoF	I_Na_/I_K_—LoF/GoF	I_Ca-L_—GoF
**Effect on action potential**	Prolongation							
**Frequency among LQTS**	30–35%	25–30%	5–10%	<1%	1–3%	<1%	<1%	<1%
**Associated syndromes**	Jervell & Lange-Nielsen	-	-	-	Jervell & Lange-Nielsen Romano-Ward	-	Andersen-Tawil	Timothy
**Inheritance**	AD	AD	AD	AD	AD/AR	AD	AD	AD
**Penetrance**	65%	80%	79%/90%	-	20%	Not documented	80–94%	Timothy syndrome: 100% Non-syndromic variants: 60–80%
**QT behaviour during exercise**	Failure to shorten	Normal	Supranormal	-	-	-	-	-
**Trigger for arrhythmic events**	Exercise (swimming)	Arousal (sudden noises)	During rest	-	-	-	-	-
**Age of onset**	Childhood	Puberty	Puberty	-	Young adulthood	-	First/Second decade	Any age
**Gender prevalence**	Male	Female	Female	-	Female	-	-	No difference
**Therapy**	Beta-blocker, ICD, LCSD		Sodium channel blocker (i.e., mexiletine), beta-blocker, ICD, LCSD	Beta-blocker, ICD, LCSD	Beta-blocker, ICD, LCSD	Beta-blocker, ICD, LCSD	Carbonic anhydrase inhibitors; oral/iv potassium; beta-blocker, ICD, LCSD	Beta-blocker, ICD, LCSD

AD: Autosomal Dominant; AR: Autosomal Recessive; GoF: Gain of Function; ICD: Implantable Cardioverter-Defibrillator; LCSD: Left Cardiac Sympathetic Denervation; LoF: Loss of Function.

**Table 2:** Modified Schwartz score for long QT syndrome diagnosis.

Findings			Points
ECG	QTc	≥480 ms	3.5
		=460–479 ms	2
		=450–459 ms (in males)	1
		≥480 ms during 4th minute of recovery from exercise stress test	1
	Torsade de pointes		2
	T wave alternans		1
	Notched T wave in 3 leads		1
	Low heart rate for age		0.5
Clinical history	Syncope	With stress	2
		Without stress	1
Family history	Family member(s) with definite LQTS		1
	Unexplained SCD at age <30 years in first-degree family		0.5
Genetic finding	Pathogenic mutation		3.5

ECG, electrocardiogram; LQTS, long QT syndrome; SCD, sudden cardiac death. Diagnosis of LQTS with a score > 3.0.

## Abbreviations

ECG	Electrocardiogram
BPM	Beats per minute
ICD	Implantable cardioverter-defibrillator
JLNS	Jervell and Lange-Nielson syndrome
LCSD	Left cardiac sympathetic denervation
LQTS	Congenital long QT syndrome
QTc	Heart rate-corrected QT interval
SIDS	Sudden infant death syndrome
